# Clinical relevance of salivary pepsin detection in diagnosing gastroesophageal reflux disease subtypes

**DOI:** 10.1093/gastro/goad053

**Published:** 2023-09-13

**Authors:** Mengyu Zhang, Tingting Wu, Niandi Tan, Songfeng Chen, Qianjun Zhuang, Yu Luo, Yinglian Xiao

**Affiliations:** Department of Gastroenterology, The First Affiliated Hospital of Sun Yat-sen University, Guangzhou, Guangdong, P. R. China; Department of Gastroenterology, The First Affiliated Hospital of Sun Yat-sen University, Guangzhou, Guangdong, P. R. China; Department of Gastroenterology, The First Affiliated Hospital of Sun Yat-sen University, Guangzhou, Guangdong, P. R. China; Department of Gastroenterology, The First Affiliated Hospital of Sun Yat-sen University, Guangzhou, Guangdong, P. R. China; Department of Gastroenterology, The First Affiliated Hospital of Sun Yat-sen University, Guangzhou, Guangdong, P. R. China; Department of Gastroenterology, The First Affiliated Hospital of Sun Yat-sen University, Guangzhou, Guangdong, P. R. China; Department of Gastroenterology, The First Affiliated Hospital of Sun Yat-sen University, Guangzhou, Guangdong, P. R. China

**Keywords:** salivary pepsin, gastroesophageal reflux disease, laryngopharyngeal reflux, screening and diagnosis

## Abstract

**Background:**

Gastroesophageal reflux disease (GERD) is heterogeneous with a varied symptom spectrum and reflux profiles. Its definite diagnosis often requires invasive tools including endoscopy or reflux monitoring. The aim of this study was to investigate the clinical relevance of salivary pepsin detection as a non-invasive screening tool to diagnose GERD of different subtypes.

**Methods:**

A total of 77 patients with suspected GERD symptoms and 12 asymptomatic controls were analysed. All participants performed symptom evaluation, upper endoscopy, esophageal manometry, and 24-hour multichannel intraluminal impedance-dual pH probe monitoring. Saliva was self-collected across three different time points: at early fasting, postprandially, and at symptom occurrence. Salivary pepsin levels were measured via Peptest. The optimal threshold of salivary pepsin for diagnosing distal or proximal reflux was determined according to a receiver-operating characteristic curve.

**Results:**

The average salivary pepsin concentration of suspected GERD patients was significantly higher than that of controls (100.63 [68.46, 141.38] vs 67.90 [31.60, 115.06] ng/mL, *P *=* *0.044), although no difference was found among patients with different symptom spectrums. The distal reflux group had a higher average pepsin concentration than non-reflux patients (170.54 [106.31, 262.76] vs 91.13 [63.35, 127.63] ng/mL, *P *=* *0.043), while no difference was observed between the distal reflux group and the proximal reflux group. The optimal cut-off value of salivary pepsin concentration for diagnosing pathological distal reflux was 157.10 ng/mL, which was higher than that for diagnosing pathological proximal reflux (122.65 ng/mL). The salivary pepsin concentration was significantly correlated with distal and proximal reflux parameters.

**Conclusions:**

Salivary pepsin measurement can help in identifying true GERD with pathological distal reflux or proximal reflux, regardless of different symptom spectrums. A higher threshold should be applied for diagnosing distal reflux than for proximal reflux.

## Introduction

Gastroesophageal reflux disease (GERD) is a common digestive disease with a pooled prevalence of 13.8% all around the world [1]. Albeit with the advances of diagnostic tests for GERD, identifying patients with GERD remains a huge dilemma in clinical practice. GERD is generally empirically diagnosed based on symptoms and response to a proton-pump inhibitor (PPI) trial. Gold-standard testing requires an invasive endoscopic procedure and ambulatory pH monitoring or pH-impedance monitoring [2, 3]. GERD is thought to be a heterogeneous disease, embodied in its varied symptom spectrum and reflux profile [4]. First, GERD patients can not only have typical esophageal symptoms but also extraesophageal symptoms, which makes the symptom-based diagnosis less reliable. In terms of the reflux profile, refluxate can reach the proximal esophagus or even the laryngopharynx, which is so-called laryngopharyngeal reflux (LPR) [5]. It is investigated that the medical expenditure per year for the diagnosis and treatment of LPR patients was five to six times higher than that of typical GERD patients [6]. Furthermore, endoscopy and ambulatory reflux monitoring often uncovers normal findings, or absence of GERD, in a majority of PPI non-responders [7, 8]. To avoid overdiagnosis or a waste of medical expenditure, reliable and non-invasive approaches are urgently needed to distinguish patients with a high or a low likelihood of GERD.

Salivary pepsin measurement has been proposed as a non-invasive method for GERD diagnosis [9, 10]. Pepsin, secreted by gastric chief cells, exists in the stomach or duodenum physiologically as a digestive enzyme. GERD is suspected when pepsin is detected in the saliva. Peptest (RD Biomed, Cottingham, UK) is a lateral flow device that can rapidly detect and quantify the concentration of salivary pepsin. Previous studies have supported the value of salivary pepsin for identifying GERD and LPR [11, 12]. Hayat *et al.* [11] and Du *et al.* [13] found that the salivary pepsin concentration in GERD patients was significantly higher than that in healthy controls. A multicenter study from the UK has found higher salivary pepsin concentrations in suspected LPR patients. Besides, previous studies have demonstrated the correlation between salivary pepsin and reflux parameters, esophageal injury severity, and the scores of reflux symptom questionnaires [12–14].

So far, it is unknown whether salivary pepsin measurement can serve as a screening test in clinical practice to identify true GERD. To address these knowledge gaps, we aimed to examine the clinical relevance of salivary pepsin to diagnose GERD among patients with different symptoms or different reflux subtypes, and to determine the optimal threshold of salivary pepsin concentration to diagnose GERD.

## Patients and methods

### Study design

Consecutive adult outpatients with suspected GERD symptoms were prospectively enrolled between May 2021 and May 2022 in the First Affiliated Hospital of Sun Yat-sen University (Guangzhou, China). Additionally, age- and gender-matched asymptomatic controls were enrolled concurrently. All participants were required to fill in three questionnaires, including a gastroesophageal reflux disease questionnaire (GerdQ) [15], reflux symptom index (RSI) [16], and a five-point symptom Likert scale to assess the frequency and severity of the symptoms. All participants had upper endoscopy, high-resolution manometry (HRM), and 24-hour multichannel intraluminal impedance-dual pH probe (dual MII-pH) monitoring performed off anti-secretory medications. Saliva was self-collected by participants across three different time points: at early fasting, postprandially, and at symptom occurrence. As for asymptomatic controls, they were required to collect only early fasting and postprandial saliva samples. Salivary pepsin levels were measured via Peptest.

The study conformed to the ethical guidelines of the Declaration of Helsinki (7th revision) and was approved by the Ethical Review Board of the First Affiliated Hospital of Sun Yat-sen University (No. [2021] 479). Written informed consent was obtained from each subject.

### Subjects

A total of 81 adult outpatients (aged 18–65 years) presented with esophageal and/or extraesophageal symptoms suspicious for GERD and 14 asymptomatic controls were prospectively enrolled and screened. Exclusion criteria for the patient group were as follows: unable to tolerate reflux monitoring; organic lesions (e.g. peptic ulcers, Barrett’s esophagus, cancer) on upper endoscopy; major motility disorders on HRM; history of upper gastroesophageal surgery; on antacids, prokinetics, PPI or potassium-competitive acid blocker 7 days before or during the esophageal function tests. For the control group, subjects with lesions on endoscopy were also excluded.

According to their symptom profile, patients were grouped as follows: (i) esophageal symptom group: patients who only had esophageal symptoms (regurgitation, heartburn); (ii) extraesophageal symptom group: patients who only had extraesophageal symptoms (hoarseness, clearing of throat, sore throat, postnasal drip, cough, asthma, etc.); (iii) mixed symptom group: patients who had both esophageal and extraesophageal symptoms.

### Upper endoscopy

All the participants had upper endoscopy performed before esophageal function tests. Participants with organic lesions (e.g. peptic ulcers, Barrett’s esophagus, cancer) were identified. The severity of reflux esophagitis (RE) was staged based on the Los Angeles (LA) classification [17].

### HRM

All the participants were required to perform HRM (Manoscan 360, Medtronic, Minneapolis, MN, USA) before the 24-hour dual MII-pH monitoring off-PPI. Pressure topography was manually analysed based on the Chicago classification criteria 4.0 [18] by two investigators using Manoview Analysis Software. Esophagogastric junction-contractile integral (EGJ-CI) was calculated [19].

### 24-Hour multichannel intraluminal impedance-dual pH probe

**Figure 1.**
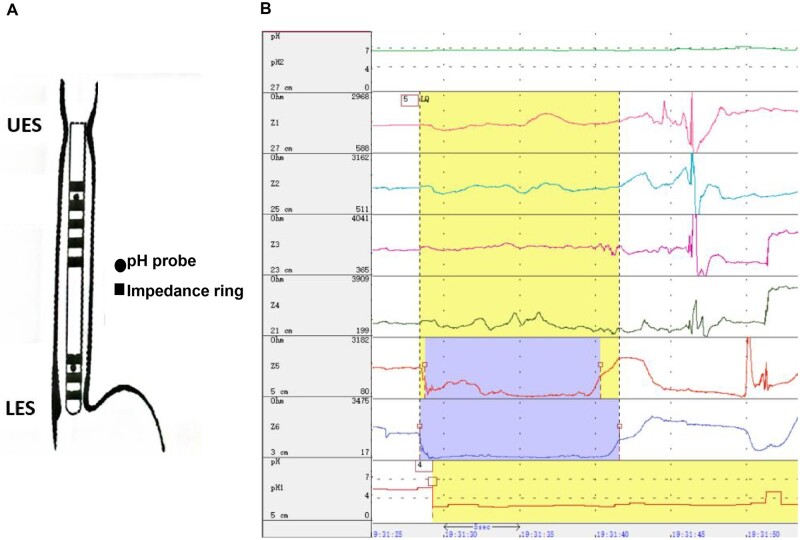
The device of multichannel intraluminal impedance-dual pH probe monitoring. UES, upper esophageal sphincter; LES, lower esophageal sphincter.

The 24-hour multichannel intraluminal impedance-dual pH probe (dual MII-pH) monitoring (Jinshan Scientific & Technology Inc., Chongqing, China) was performed off-therapy using a catheter (JSIpC-8Z2P-26) with two pH electrodes and six impedance channels. The distal pH electrode was positioned at 5 cm above the lower esophageal sphincter (LES) and the proximal one was positioned at 27 cm above the LES (Figure 1). The impedance channels were positioned at 3, 5, 21, 23, 25, and 27 cm above the LES separately. All the participants were taught to use the digital data device to record symptoms, meals, and postures during monitoring.

The data were analysed manually by two investigators following the Wingate Consensus [20] using the OMOM Analysis Software (Jinshan Scientific & Technology Inc., Chongqing, China). Meal periods were excluded from the analysis. The following dual MII-pH parameters were analysed: (i) distal reflux parameters: acid exposure time (AET), number of distal reflux episodes, post-reflux swallow-induced peristaltic wave (PSPW) index, and mean nocturnal baseline impedance (MNBI); (ii) proximal reflux parameters: time percentage under different pH value (pH<4.0, 4.5, 5.0, 5.5, 6.0, and 6.5), and number of proximal reflux episodes.

### Definition of different reflux subtypes

All suspected GERD patients were grouped after a comprehensive assessment of upper endoscopy, HRM, and dual MII-pH findings.

- Distal reflux group: based on the Lyon consensus [21], patients were required to meet at least one of the following criterion: (a) AET of >6% or LA grade C/D RE or long segment Barrett’s mucosa or peptic stricture; (b) LA grade A/B esophagitis or AET 4%–6%, and had at least one abnormal adjunctive supportive evidence: (1) number of total reflux episodes of >80; (2) low MNBI (<2,292 mmHg) and low PSPW index (≤61%); (3) positive reflux symptom association; (4) low EGJ-CI or hiatal hernia or ineffective esophageal motility.
- Proximal reflux group: patients were required to meet at least one of the criteria as described in a previous study [22]: (a) time percentage when pH of <4.0 was >0.02%; (b) time percentage when pH of <5.0 was >2.33%; (c) time percentage when pH of <6.0 was >21.41%.
- Non-reflux group: patients who did not meet the criteria of the distal reflux and proximal reflux groups.

Among all the included suspected GERD patients, the distal reflux group and the proximal reflux group were considered as having true GERD, while the non-reflux group were patients without pathological reflux.

### Saliva sample collection and analysis

Saliva was self-collected by participants across three different time points: at early fasting, postprandially (1–2 hours after a meal), and at symptom occurrence (within 15 mins), during the day when the 24-hour dual MII-pH monitoring was performed. These saliva samples were carried back the next day when participants returned to the hospital to remove the MII-pH device. The saliva samples were kept in cold storage with an ice pack during transportation.

Saliva samples were placed into 15-mL sterile plastic tubes containing 0.5 mL of 0.01 mol/L citric acid at pH 2.5 and then promptly transferred to a refrigerator at 4°C. Pepsin was detected and measured using the Peptest (RD Biomed, Cottingham, UK) within 7 days of collection.

The protocol for pepsin detection was as follows: (i) take a 500-μL saliva sample and centrifuge at a speed of 4,000 rpm for 5 mins; (ii) mix 80 μL of supernatants with 240 μL of migration buffer solution and perform vortex agitation for 10 s; (iii) take 80 μL of the solution into the lateral flow device. Transfer the lateral flow device to the Peptest recorder, which provides a quantified concentration of pepsin in ng/mL. Peptest can detect pepsin concentrations in the range of 16–500 g/mL. Salivary pepsin concentrations at three different time points (at early fasting, postprandially, and at symptom occurrence) were recorded for each subject, and the average and maximum concentrations were also calculated based on those at the three time points.

### Statistical analysis

Normally distributed variables are presented as mean±standard deviation (SD); non-normally distributed variables are presented as median and interquartile range (IQR); categorical variables are presented as numbers and percentage. Grouped data were compared using the two-tailed Student’s *t*-test, One-Way Analysis of Variance (ANOVA) test, Mann–Whitney *U* test, or Kruskal–Wallis test, when appropriate. Categorical data were compared using the χ^2^ test or Fisher’s exact test, when appropriate. A receiver-operating characteristic (ROC) curve was performed to obtain the cut-off value of salivary pepsin detection for identifying GERD subgroups. The sensitivity (SEN), specificity (SPE), true positive (TP), false positive (FP), false negative (FN), and true negative (TN) were calculated. Spearman rank correlation was used to evaluate the correlation between salivary pepsin and different parameters. Differences were considered statistically significant when *P* < 0.05. All the statistical analyses were conducted using the SPSS 22.0 (IBM, Armonk, NY, USA).

## Results

### Baseline and clinical characteristics

**Figure 2.**
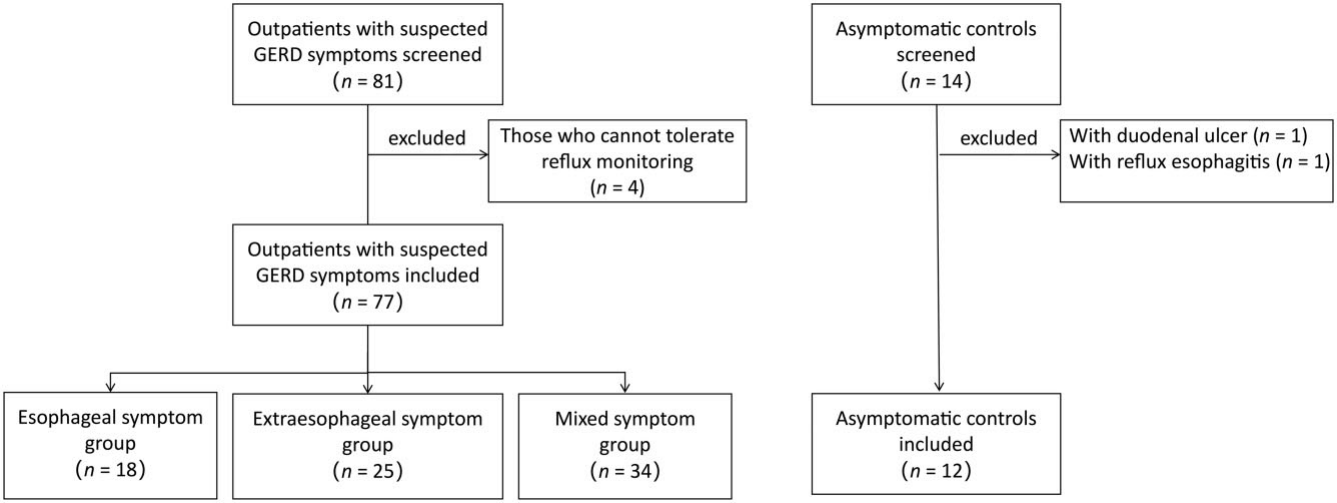
The flow chart of the study. GERD, gastroesophageal reflux disease.

Of 81 patients with suspected GERD symptoms, 4 patients who could not tolerate reflux monitoring were excluded. Of 14 asymptomatic controls, 2 (1 with duodenal ulcer, 1 with LA-B grade RE) were excluded. Altogether 77 patients and 12 asymptomatic controls were included in the final analysis (Figure 2).

Among the included patients, 18 patients had only esophageal symptoms, 25 had only extraesophageal symptoms, and 34 had mixed symptoms. As shown in Table 1, there were no significant differences in age, gender proportion, body mass index (BMI), and life habits among the three different symptom groups. We found 10 (13.0%) cases of RE, all of which were LA-A or LA-B grades. Five patients had hiatal hernia on HRM. No significant difference of EGJ-CI was observed among the groups. The extraesophageal symptom group had significantly higher upright AET than the control group did (1.00 vs 0.10, *P *=* *0.013), and all three symptom groups had significantly more reflux episodes than controls did. However, no significant differences in AET and the number of reflux episodes were found among the three symptom groups. Also, no significant differences in MNBI, PSPW index, and proximal reflux parameters were observed among the groups. Based on a comprehensive assessment of upper endoscopy, HRM, and dual MII-pH findings, patients were further divided into three reflux subtypes: distal reflux group (*n *=* *10), proximal reflux group (*n *=* *8), and non-reflux group (*n *=* *59).

**Table 1.** Characteristics of the 89 included subjects

Parameter	Esophageal symptom group (*n *=* *18)	Extraesophageal symptom group (*n *=* *25)	Mixed symptom group (*n *=* *34)	Control group (*n *=* *12)	*P*-value
Baseline characteristic					
Age, years, mean ± SD	48.0 ± 14.3	42.2 ± 12.0	43.1 ± 11.7	37.3 ± 9.8	0.078
Male, *n* (%)	8 (44.4)	14 (56.0)	16 (47.1)	3 (25.0)	0.115
BMI, kg/m^2^, mean ± SD	21.81 ± 4.32	21.48 ± 2.92	21.99 ± 3.70	21.50 ± 3.59	0.793
Drinking, *n* (%)	5 (27.8)	7 (28.0)	9 (26.5)	2 (16.7)	0.670
Smoking, *n* (%)	3 (16.7)	4 (16.0)	3 (8.8)	0 (0.0)	0.404
Coffee consumption, *n* (%)	3 (16.7)	1 (4.0)	5 (14.7)	4 (33.3)	0.169
GerdQ score, median (IQR)	10.5 (9.0, 13.5)^a^,^c^	6.0 (4.5, 6.0)	10.0 (9.0, 12.0)^a^,^c^	6.0 (4.5, 6.0)	<0.001
Reflux symptom index score, median (IQR)	4.0 (3.00, 5.00)	10.0 (6.50, 14.00)^b^,^c^	10.0 (6.75, 16.00)^b^,^c^	0.0 (0.00, 0.00)	<0.001
Upper endoscopy					
Reflux esophagitis, *n* (%)	2 (11.1)	3 (12.0)	5 (14.7)	0 (0.0)	0.321
LA-A	1 (5.6)	1 (4.0)	4 (11.8)	0 (0.0)	–
LA-B	1 (5.6)	2 (8.0)	1 (2.9)	0 (0.0)	–
HRM parameter					
Hiatal hernia, *n* (%)	2 (11.1)	1 (4.0)	2 (5.9)	0 (0.0)	–
EGJ-CI, mmHg·cm, median (IQR)	42.88 (25.88, 81.50)	40.49 (23.88, 49.61)	41.90 (27.77, 65.06)	53.52 (42.83, 72.24)	0.069
Dual MII-pH: distal parameter					
Upright AET, median (IQR)	0.85 (0.20, 4.80)	1.00 (0.70, 2.80)^c^	0.85 (0.20, 1.80)	0.10 (0.00, 0.30)	0.018
Supine AET, median (IQR)	0.10 (0.00, 1.30)	0.00 (0.00, 0.20)	0.00 (0.00, 0.30)	0.00 (0.00, 0.00)	0.122
Total AET, median (IQR)	0.55 (0.10, 3.70)	0.80 (0.40, 1.20)	0.50 (0.10, 1.50)	0.05 (0.00, 0.70)	0.069
Number of acid reflux episodes, median (IQR)	16.5 (9.0, 37.0)^c^	16.0 (9.0, 28.0)^c^	13.5 (4.0, 31.0)^c^	2.0 (1.0, 5.5)	0.010
Number of total reflux episodes, median (IQR)	25.5 (16.0, 47.0)^c^	27.0 (14.0, 40.0)^c^	20.0 (11.0, 47.0)^c^	7.0 (3.0, 16.5)	0.013
Number of liquid reflux episodes, median (IQR)	8.0 (4.0, 16.0)^c^	6.0 (2.0, 12.0)^c^	6.5 (2.0, 11.0)^c^	0.5 (0.0, 2.5)	0.003
Number of mixed reflux episodes, median (IQR)	20.0 (7.0, 31.0)	18.0 (8.0, 29.0)	14.5 (7.0, 33.0)	5.5 (3.0, 14.0)	0.098
MNBI, median (IQR)	1,547 (911, 3,219)	1,056 (792, 3,866)	1,370 (1,091, 3,098)	1,899 (1,468, 3,987)	0.078
PSPW index, median (IQR)	0.55 (0.34, 0.78)	0.49 (0.42, 0.75)	0.51 (0.28, 0.69)	0.67 (0.31, 0.82)	0.083
Dual MII-pH: proximal parameter					
Time percentage at pH < 4.0, median (IQR)	0.0 (0.0, 0.0)	0.0 (0.0, 0.0)	0.0 (0.0, 0.0)	0.0 (0.0, 0.0)	0.655
Time percentage at pH < 4.5, median (IQR)	0.0 (0.0, 0.0)	0.0 (0.0, 0.0)	0.0 (0.0, 0.0)	0.0 (0.0, 0.0)	0.655
Time percentage at pH < 5.0, median (IQR)	0.0 (0.0, 0.0)	0.0 (0.0, 0.0)	0.0 (0.0, 0.0)	0.0 (0.0, 0.0)	0.233
Time percentage at pH < 5.5, median (IQR)	0.0 (0.0, 1.0)	0.0 (0.0, 0.0)	0.0 (0.0, 0.0)	0.0 (0.0, 1.0)	0.609
Time percentage at pH < 6.0, median (IQR)	1.5 (0.0, 15.0)	0.0 (0.0, 11.0)	1.0 (0.0, 10.0)	0.5 (0.0, 1.5)	0.448
Time percentage at pH < 6.5, median (IQR)	21.0 (7.0, 36.0)	12.0 (1.0, 37.0)	14.0 (8.0, 26.0)	8.0 (1.5, 18.5)	0.733
Number of proximal reflux episodes, median (IQR)	1.0 (0.0, 4.0)	1.0 (0.0, 4.0)	1.0 (0.0, 2.0)	0.0 (0.0, 0.0)	0.113

AET, acid exposure time; BMI, body mass index; dual MII-pH, multichannel intraluminal impedance-dual pH; EGJ-CI, esophagogastric junction-contractile integral; GerdQ, gastroesophageal reflux disease questionnaire; HRM, high-resolution manometry; IQR, interquartile range; LA, Los Angeles; MNBI, mean nocturnal baseline impedance; PSPW, post-reflux swallow-induced peristaltic wave; SD, standard deviation.

a Compared with extraesophageal symptom group, *P*<0.05.

b Compared with esophageal symptom group, *P*<0.05.

c Compared with control group, *P*<0.05.

### Clinical relevance of salivary pepsin measurement to identify GERD

#### Patients with suspected GERD symptoms vs asymptomatic controls

Patients with suspected GERD symptoms had significantly higher average and higher maximum salivary pepsin concentrations than asymptomatic controls did (100.63 [68.46, 141.38] vs 67.90 [31.60, 115.06] ng/mL, *P *=* *0.044; 144.60 [118.00, 221.60] vs 111.80 [54.33, 138.58] ng/mL, *P *=* *0.015, respectively; Table 2). Also, the postprandial salivary pepsin concentration in suspected GERD patients was significantly higher than that in asymptomatic controls (118.75 [66.38, 148.10] vs 55.35 [27.09, 101.01] ng/mL, *P *=* *0.014).

**Table 2.** The comparison of salivary pepsin concentrations among different symptom groups

Time	Esophageal symptom group	Extraesophageal symptom group	Mixed symptom group	Control group	*P*-value
Postprandial	131.10 (64.74, 194.00)	126.10 (49.83, 164.80)	103.95 (69.25, 136.90)	55.35 (27.09, 101.01)	0.072
Early fasting	36.65 (16.00, 182.90)	94.30 (0.00, 132.80)	78.00 (0.00, 127.30)	82.70 (0.00, 120.25)	0.985
Symptom occurrence	111.15 (16.00, 216.93)	114.50 (45.60, 188.50)	118.50 (73.40, 143.20)	–	0.929
Maximum	154.25 (114.93, 223.45)	180.00 (121.35, 246.60)	136.55 (117.40, 204.23)	111.80 (54.33, 138.58)	0.049
Average	101.02 (61.27, 147.41)	123.30 (65.00, 161.34)	91.95 (71.00, 129.71)	67.90 (31.60, 115.06)	0.165

#### Comparison among suspected GERD patients with different symptoms

As shown in Table 2, significant difference in the maximum salivary pepsin concentrations can be found among different symptom subgroups and the control group. However, no significant difference was found in further pairwise comparisons.

#### True GERD patients vs non-reflux patients vs controls

**Figure 3.**
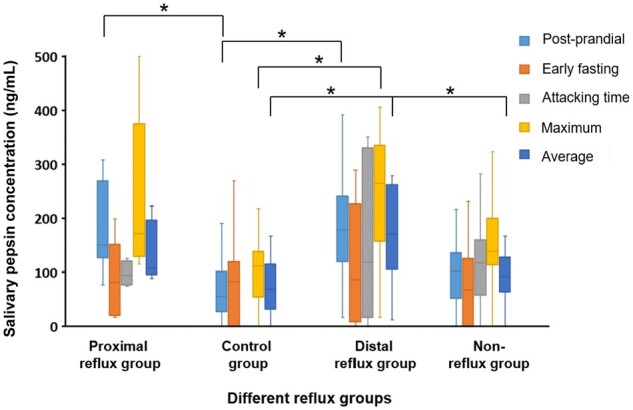
The salivary pepsin concentration of different reflux groups and control group. *P-value < 0.05.

There were significant differences in the maximum and average salivary pepsin concentrations among the different reflux subgroups and the control group. Pairwise comparisons are shown in Figure 3.

When compared with the control group, the distal reflux group had higher maximum salivary pepsin concentrations (264.05 [157.30, 335.53] vs 111.80 [54.33, 138.58] ng/mL, *P *=* *0.010), higher average salivary pepsin concentrations (170.54 [106.31, 262.76] vs 67.90 [31.60, 115.06] ng/mL, *P *=* *0.009), and higher postprandial salivary pepsin concentrations (178.95 [119.48, 240.98] vs 55.35 [27.09, 101.01] ng/mL, *P *=* *0.005). Besides, the proximal reflux group also had higher postprandial salivary pepsin concentrations than the control group did (150.08 [126.65, 268.81] vs 55.35 [27.09, 101.01] ng/mL, *P *=* *0.009).

When compared with the non-reflux group, the distal reflux group had significantly higher average salivary pepsin concentrations (170.54 [106.31, 262.76] vs 91.13 [63.35, 127.63] ng/mL, *P *=* *0.043). However, no significant difference in salivary pepsin concentrations was observed between the proximal reflux group and the non-reflux group or between the distal reflux group and the proximal reflux group.

### Optimal threshold of salivary pepsin concentration to diagnose GERD

**Figure 4.**
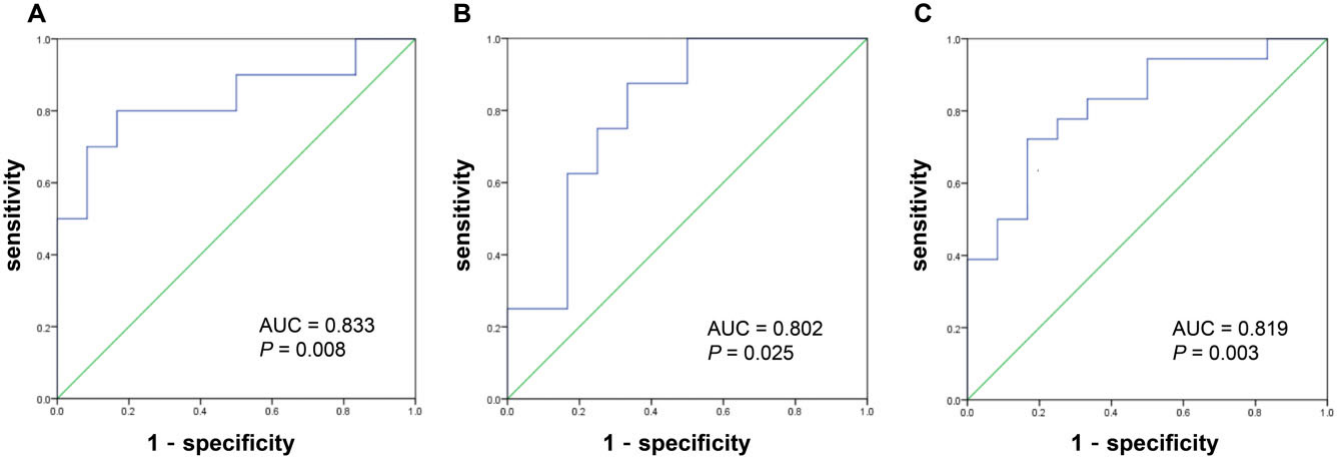
Receiver-operating characteristic curve of salivary pepsin detection for diagnosing gastroesophageal reflux disease of different reflux subtypes. (A) The cut-off value of the salivary pepsin concentration for diagnosis of distal reflux was 157.10 ng/mL. (B) The cut-off value of the salivary pepsin concentration for diagnosis of proximal reflux was 122.65 ng/mL. (C) The cut-off value of the salivary pepsin concentration for diagnosis of distal or proximal reflux was 148.10 ng/mL.

As shown in Figure 4, the ROC analysis was conducted to determine the cut-off value for salivary pepsin detection to diagnose patients with pathological distal or proximal reflux, using the maximum salivary pepsin concentration as the random Peptest detection value in clinical practice.

The optimal cut-off value of the salivary pepsin concentration to identify the distal reflux group was 157.10 ng/mL, with the SEN of 80.00%, the SPE of 83.58%, the TP of 8, the FP of 11, the TN of 56, and the FN of 2. The optimal cut-off value of the salivary pepsin concentration to identify the proximal reflux group was 122.65 ng/mL, with the SEN of 100.00% and the SPE of 66.67%, the TP of 8, the FP of 23, the TN of 46, and the FN of 0. To identify patients with either distal reflux or proximal reflux, the cut-off value of the salivary pepsin concentration was 148.10 ng/mL, with the SEN of 72.22% and the SPE of 83.05%, the TP of 13, the FP of 10, the TN of 49, and the FN of 5.

### Correlation analysis of salivary pepsin concentration

In terms of the correlation between the salivary pepsin and baseline characteristics, patients with long-term consumption of coffee had significantly higher salivary pepsin concentrations at early fasting (125.80 [107.40, 150.90] vs 58.00 [0, 115.50] ng/mL, *P *=* *0.003) and at symptom occurrence (185.45 [129.15, 250.70] vs 104.70 [57.40, 142.90] ng/mL, *P *=* *0.022) than those who did not. A similar result was found in the average salivary pepsin concentrations (134.28 [100.35, 161.63] vs 91.13 [62.08, 126.15] ng/mL, *P *=* *0.033). There was no significant correlation between salivary pepsin and gender, age, BMI, GerdQ score, or RSI score.

As shown in Table 3, significant correlation was seen between salivary pepsin concentrations and reflux parameters. In terms of distal reflux parameters, total AET and upright AET were positively correlated with the average and postprandial salivary pepsin concentrations. The number of liquid reflux episodes was positively correlated with the average and early fasting salivary pepsin concentrations. The PSPW index was negatively correlated with the postprandial salivary pepsin concentration. In terms of proximal reflux parameters, time percentages when the pH was <5.0, 5.5, 6.0, and 6.5 were positively correlated with salivary pepsin concentrations.

**Table 3.** Correlation between reflux parameters and salivary pepsin concentrations

Parameter	Postprandial		Early fasting		Symptom occurrence		Maximum		Average	
	*r*	*P*-value	*r*	*P*-value	*r*	*P*-value	*r*	*P*-value	*r*	*P*-value
Distal reflux parameter										
Upright AET	0.230	0.029	0.012	0.278	0.021	0.127	0.181	0.065	0.228	0.029
Total AET	0.231	0.027	0.113	0.112	0.107	0.471	0.088	0.109	0.229	0.029
Number of liquid reflux episodes	0.101	0.081	0.238	0.031	0.084	0.810	0.104	0.165	0.217	0.041
PSPW index	–0.217	0.041	0.200	0.348	0.181	0.538	0.241	0.098	0.102	0.253
Proximal reflux parameter										
Time percentage at pH < 5.0	0.269	0.011	0.602	0.001	0.199	0.691	0.635	0.011	0.880	0.001
Time percentage at pH < 5.5	0.281	0.008	0.051	0.541	0.217	0.372	0.110	0.415	0.309	0.479
Time percentage at pH < 6.0	0.383	0.001	0.109	0.287	0.076	0.685	0.222	0.037	0.321	0.002
Time percentage at pH < 6.5	0.416	0.001	0.243	0.028	0.374	0.291	0.273	0.010	0.417	0.001

AET, acid exposure time; PSPW, post-reflux swallow-induced peristaltic wave.

## Discussion

Given the fact that GERD is a heterogeneous disease that presents with a varied symptom spectrum and reflux profiles, its definite diagnosis often requires a comprehensive assessment of a PPI trial and invasive tools including endoscopy or reflux monitoring [23, 24]. Therefore, a non-invasive screening tool is critically needed in the primary care setting to distinguish patients with high or low likelihood of GERD. This study demonstrated that suspected GERD patients had significantly higher salivary pepsin concentrations than asymptomatic controls, although no difference was found among patients with different symptom spectrums. Salivary pepsin measurement can help in screening and identifying true GERD, including pathological distal and proximal reflux. A higher threshold of salivary pepsin level should be required to diagnose pathological distal reflux than to diagnose pathological proximal reflux. The salivary pepsin level is correlated with distal and proximal reflux parameters.

In clinical practice, GERD is empirically screened and diagnosed based on typical esophageal symptoms (heartburn and regurgitation) [2]. However, some GERD patients can present with only extraesophageal symptoms or mixed symptoms [25]. Salivary pepsin measurement is now considered a promising non-invasive screening tool for GERD, among either patients with pure typical esophageal symptoms or patients with pure extraesophageal symptoms. As for patients with only typical esophageal symptoms, significantly higher salivary pepsin concentrations are observed in GERD patients than in non-GERD patients and asymptomatic controls [11, 13]. As for patients with only extraesophageal symptoms, it was found that LPR patients had higher salivary pepsin concentrations than asymptomatic controls did [26, 27]. However, most of these studies were conducted among patients with typical GERD symptoms alone or LPR symptoms alone, while our study was conducted among suspected GERD patients with heterogeneous symptoms, including those with mixed symptoms, which was more similar to the real-world setting. Given the complex symptom spectrum of GERD, it raised a clinical question of whether salivary pepsin concentrations differ among heterogeneous symptom groups. Our results found that suspected GERD patients did have significantly higher salivary pepsin concentrations than asymptomatic controls. However, no difference in salivary pepsin concentrations was found among different symptom groups, which can be partly explained by the complex mechanism of symptom perception. Thus, salivary pepsin detection cannot simply distinguish different symptom groups during the empirical symptom-based diagnosis of GERD. The definite diagnosis of GERD requires a comprehensive assessment of upper endoscopy, HRM, and dual MII-pH findings. In our study, we then separated suspected GERD patients into different reflux subtypes and found significantly higher salivary pepsin concentrations in both the distal reflux group and the proximal reflux group than in asymptomatic controls. Of note, the distal reflux group had significantly higher salivary pepsin concentrations than non-reflux patients, which proves that salivary pepsin detection can help in distinguishing true GERD from non-GERD patients and controls. However, no significant difference was found between the distal reflux group and the proximal reflux group.

In our study, the optimal threshold of salivary pepsin for identifying GERD was determined via ROC analysis. It was found that the optimal cut-off value of the salivary pepsin concentration for diagnosing pathological distal reflux was 157.10 ng/mL, which is higher than that for diagnosing pathological proximal reflux (122.65 ng/mL). It was a controversial issue concerning the difference in salivary pepsin detection between the distal reflux group and the proximal reflux group. Some researchers found that salivary pepsin concentrations were higher in GERD patients than in LPR patients [12], while Bor *et al*. [28] found that the positive rate of Peptest was similar in GERD and LPR patients (72.7% vs 65.2%, *P *>* *0.05). In fact, pepsin plays a more important role in the reflux occurring in proximal esophagus instead of that which occurs in distal esophagus [29]. Proximal esophagus is characterized with a more alkalic environment and the proteolytic activity of pepsin is maintained up to a pH of 6 [30]. What is more, proximal esophageal innervation is more superficial than in the distal esophagus [31], suggesting that the proximal esophagus has a more susceptible anti-reflux barrier and is thereby prone to injury with lower levels of pepsin. Further research is required to investigate the utility of salivary pepsin detection in diagnosing pathological distal reflux and pathological proximal reflux.

Our study also demonstrated the significant correlation of salivary pepsin concentrations with reflux parameters, including AET, the number of distal reflux episodes, and time percentages when the proximal pH is <5.0, 5.5, 6.0, and 6.5, which was consistent with previous studies [11, 13]. Additionally, it was shown that subjects with a coffee-drinking history had significantly higher salivary pepsin concentrations, though confounding factors cannot be excluded. Previous study has also found that cigarette and food intake had an influence on salivary pepsin concentrations [32, 33]. Ascertaining the influence factors may be necessary to improve the credibility of salivary pepsin detection.

This study had some limitations. First, patients were recruited based on symptoms only and many extraesophageal symptoms can be unspecific. The proportion of pathological reflux cases was low, which may lead to bias in statistical analysis. Second, the diagnostic standard of pathological proximal reflux that we used in the study was referred to a previous study, in which the catheter was not from the same manufacturer as ours, which may have led to bias in diagnosis. Third, we should pay attention to the variability of salivary pepsin detection via Peptest. Researchers have mentioned time dependence and low repeatability in the same sample and the same case [34]. It asked for a more accurate and standardized operating procedure in salivary pepsin detection. Our study reduced detection error through multiple sampling.

## Conclusions

Salivary pepsin detection is a non-invasive and accessible screening tool to identify true GERD patients with pathological distal reflux or proximal reflux, regardless of different symptom spectrums. A higher salivary pepsin concentration threshold should be applied to diagnose pathological distal reflux. Salivary pepsin concentrations are correlated with both distal and proximal reflux parameters.

## Authors’ Contributions

M.Z. and T.W. were responsible for data analysis and interpretation as well as the first draft of the manuscript; N.T., S.C., Q.Z., and Y.L. were responsible for data analysis; Y.X. was responsible for the conception and design as well as the critical revision of the article. All authors have read and approved the final version of the manuscript.

## References

[1] Eusebi LH , Ratnakumaran R, Yuan Y et al Global prevalence of, and risk factors for, gastro-oesophageal reflux symptoms: a meta-analysis. Gut 2018;67:430–40. 28232473 10.1136/gutjnl-2016-313589

[2] Katz PO , Dunbar KB, Schnoll-Sussman FH et al ACG clinical guideline for the diagnosis and management of gastroesophageal reflux disease. Am J Gastroenterol 2022;117:27–56. 34807007 10.14309/ajg.0000000000001538 PMC8754510

[3] Xiao YL , Zhou LY, Hou XH et al; Chinese Society of Gastroenterology. Chinese expert consensus on gastroesophageal reflux disease in 2020. J Dig Dis 2021;22:376–89. 34105263 10.1111/1751-2980.13028

[4] Savarino E , Zentilin P, Savarino V. NERD: an umbrella term including heterogeneous subpopulations. Nat Rev Gastroenterol Hepatol 2013;10:371–80. 23528345 10.1038/nrgastro.2013.50

[5] Barrett CM , Patel D, Vaezi MF. Laryngopharyngeal reflux and atypical gastroesophageal reflux disease. Gastrointest Endosc Clin N Am 2020;30:361–76. 32146951 10.1016/j.giec.2019.12.004

[6] Francis DO , Rymer JA, Slaughter JC et al High economic burden of caring for patients with suspected extraesophageal reflux. Am J Gastroenterol 2013;108:905–11. 23545710 10.1038/ajg.2013.69

[7] Delshad SD , Almario CV, Chey WD et al Prevalence of gastroesophageal reflux disease and proton pump inhibitor-refractory symptoms. Gastroenterology 2020;158:1250–61 e2. 31866243 10.1053/j.gastro.2019.12.014 PMC7103516

[8] Abdallah J , George N, Yamasaki T et al Most patients with gastroesophageal reflux disease who failed proton pump inhibitor therapy also have functional esophageal disorders. Clin Gastroenterol Hepatol 2019;17:1073–80 e1. 29913281 10.1016/j.cgh.2018.06.018

[9] Hayat JO , Yazaki E, Moore AT et al Objective detection of esophagopharyngeal reflux in patients with hoarseness and endoscopic signs of laryngeal inflammation. J Clin Gastroenterol 2014;48:318–27. 24172180 10.1097/MCG.0000000000000011

[10] Li YW , Sifrim D, Xie C et al Relationship between salivary pepsin concentration and esophageal mucosal integrity in patients with gastroesophageal reflux disease. J Neurogastroenterol Motil 2017;23:517–25. 28992675 10.5056/jnm16178 PMC5628983

[11] Hayat JO , Gabieta-Somnez S, Yazaki E et al Pepsin in saliva for the diagnosis of gastro-oesophageal reflux disease. Gut 2015;64:373–80. 24812000 10.1136/gutjnl-2014-307049

[12] Spyridoulias A , Lillie S, Vyas A et al Detecting laryngopharyngeal reflux in patients with upper airways symptoms: symptoms, signs or salivary pepsin? Respir Med 2015;109:963–9. 26044812 10.1016/j.rmed.2015.05.019

[13] Du X , Wang F, Hu Z et al The diagnostic value of pepsin detection in saliva for gastro-esophageal reflux disease: a preliminary study from China. BMC Gastroenterol 2017;17:107. 29041918 10.1186/s12876-017-0667-9 PMC5645897

[14] Weitzendorfer M , Antoniou SA, Schredl P et al Pepsin and oropharyngeal pH monitoring to diagnose patients with laryngopharyngeal reflux. Laryngoscope 2020;130:1780–6. 31603541 10.1002/lary.28320 PMC7318637

[15] Bai Y , Du Y, Zou D et al; Chinese GerdQ Research Group. Gastroesophageal Reflux Disease Questionnaire (GerdQ) in real-world practice: a national multicenter survey on 8065 patients. J Gastroenterol Hepatol 2013;28:626–31. 23301662 10.1111/jgh.12125

[16] Belafsky PC , Postma GN, Koufman JA. Validity and reliability of the reflux symptom index (RSI). J Voice 2002;16:274–7. 12150380 10.1016/s0892-1997(02)00097-8

[17] Lundell LR , Dent J, Bennett JR et al Endoscopic assessment of oesophagitis: clinical and functional correlates and further validation of the Los Angeles classification. Gut 1999;45:172–80. 10403727 10.1136/gut.45.2.172 PMC1727604

[18] Yadlapati R , Kahrilas PJ, Fox MR et al Esophageal motility disorders on high-resolution manometry: Chicago classification version 4.0((c. )). Neurogastroenterol Motil 2021;33:e14058. 33373111 10.1111/nmo.14058 PMC8034247

[19] Nicodeme F , Pipa-Muniz M, Khanna K et al Quantifying esophagogastric junction contractility with a novel HRM topographic metric, the EGJ-contractile integral: normative values and preliminary evaluation in PPI non-responders. Neurogastroenterol Motil 2014;26:353–60. 24460814 10.1111/nmo.12267 PMC4605557

[20] Gyawali CP , Rogers B, Frazzoni M et al Inter-reviewer variability in interpretation of pH-impedance studies: the Wingate consensus. Clin Gastroenterol Hepatol 2021;19:1976–8 e1. 32890752 10.1016/j.cgh.2020.09.002

[21] Gyawali CP , Kahrilas PJ, Savarino E et al Modern diagnosis of GERD: the Lyon consensus. Gut 2018;67:1351–62. 29437910 10.1136/gutjnl-2017-314722 PMC6031267

[22] Sun G , Muddana S, Slaughter JC et al A new pH catheter for laryngopharyngeal reflux: normal values. Laryngoscope 2009;119:1639–43. 19504553 10.1002/lary.20282

[23] Lata T , Trautman J, Townend P et al Current management of gastro-oesophageal reflux disease—treatment costs, safety profile, and effectiveness: a narrative review. Gastroenterol Rep (Oxf) 2023;11:goad008. 37082451 10.1093/gastro/goad008 PMC10112961

[24] Hurr TJ. The six-question Gastroesophageal Reflux Disease Questionnaire (GerdQ) cannot accurately quantify reflux and reflux-associated symptoms frequency. Gastroenterol Rep (Oxf) 2022;10:goac043. 35991688 10.1093/gastro/goac043 PMC9390063

[25] Richter JE , Rubenstein JH. Presentation and epidemiology of gastroesophageal reflux disease. Gastroenterology 2018;154:267–76. 28780072 10.1053/j.gastro.2017.07.045 PMC5797499

[26] Zhang M , Chia C, Stanley C et al Diagnostic utility of salivary pepsin as compared with 24-hour dual pH/impedance probe in laryngopharyngeal reflux. Otolaryngol Head Neck Surg 2021;164:375–80. 32895009 10.1177/0194599820951183

[27] Zelenik K , Hrankova V, Vrtkova A et al Diagnostic value of the peptest(TM) in detecting laryngopharyngeal reflux. J Clin Med 2021;10:1–9. 10.3390/jcm10132996 PMC8268930 34279479

[28] Bor S , Capanoglu D, Vardar R et al Validation of peptest in patients with gastro-esophageal reflux disease and laryngopharyngeal reflux undergoing impedance testing. J Gastrointestin Liver Dis 2019;28:383–7. 31826051 10.15403/jgld-335

[29] Divakaran S , Rajendran S, Thomas RM et al Laryngopharyngeal reflux: symptoms, signs, and presence of pepsin in saliva: a reliable diagnostic triad. Int Arch Otorhinolaryngol 2021;25:e273–8. 33968232 10.1055/s-0040-1709987 PMC8096499

[30] Johnston N , Wells CW, Samuels TL et al Pepsin in nonacidic refluxate can damage hypopharyngeal epithelial cells. Ann Otol Rhinol Laryngol 2009;118:677–85. 19810610 10.1177/000348940911800913

[31] Woodland P , Aktar R, Mthunzi E et al Distinct afferent innervation patterns within the human proximal and distal esophageal mucosa. Am J Physiol Gastrointest Liver Physiol 2015;308:G525–31. 25573174 10.1152/ajpgi.00175.2014 PMC4360043

[32] Wang YF , Yang CQ, Chen YX et al Validation in China of a non-invasive salivary pepsin biomarker containing two unique human pepsin monoclonal antibodies to diagnose gastroesophageal reflux disease. J Dig Dis 2019;20:278–87. 31090180 10.1111/1751-2980.12783 PMC6851552

[33] Lechien JR , Bobin F, Muls V et al Saliva pepsin concentration of laryngopharyngeal reflux patients is influenced by meals consumed before the samples. Laryngoscope 2021;131:350–9. 32510588 10.1002/lary.28756

[34] Dolina J , Konečný Š, Ďurč P et al Evaluation of important analytical parameters of the peptest immunoassay that limit its use in diagnosing gastroesophageal reflux disease. J Clin Gastroenterol 2019;53:355–60. 29863588 10.1097/MCG.0000000000001066

